# Calcium chloride and 1-methylcyclopropene treatments delay postharvest and reduce decay of New Queen melon

**DOI:** 10.1038/s41598-019-49820-8

**Published:** 2019-09-19

**Authors:** Qiang Zhang, Wenting Dai, Xinwen Jin, Jixin Li

**Affiliations:** 1 College of Life Science and Technology, Xinjiang University, Urumqi, 830046 China; 2 Institute of Agro-products Processing Science and Technolog, Xinjiang Academy of Agricultural and Reclamation Science, Shihezi, 832000 China

**Keywords:** Molecular engineering in plants, Plant molecular biology, Plant physiology

## Abstract

In this study, newly harvested New Queen melons were treated with calcium chloride (CaCl_2_) and 1-methylcyclopropene (1-MCP) alone or in combination before storage. The results showed that the respiration rate, ethylene release, the activity and gene expression of pectinases such as polygalacturonase (PG), pectin methylesterase (PME) and pectate lyase (PL) in New Queen melons were dramatically decreased by treatments with 0.18 mol/L CaCl_2_ and/or 1 μL/L 1-MCP. Meanwhile, the climacteric behavior and flesh hardness reduction were inhibited. We also found that softer melon flesh was more conducive to the growth and reproduction of decay-causing microorganisms according to their growth curves in melons that were different in flesh hardness, suggesting inhibiting fruit softening can slow down the growth of microorganisms in fruit flesh, and thus reduce fruit decay rate. The combined use of CaCl_2_ and 1-MCP was more effective in suppressing respiration rate, ethylene release and protopectin hydrolysis, which could greatly delay the softening, reduce the decay rate, and extend the shelf life of New Queen melons.

## Introduction

Xinmi No. 13 is an elite think-rinded cultivar of New Queen melon obtained after many generations of breeding and selection, and has now become one of the popular Xinjiang melons, because of its good fruit appearance, high commercial value and unique flavor. However, postharvest ripening and softening seriously reduce the quality and shorten the shelf life of New Queen melons.

New Queen melon is a climacteric variety, characterized by a dramatic rise in respiration rate and ethylene release after harvest, while the fruit matures and softens rapidly^1^. During fruit softening, protopectin is hydrolyzed into pectin that is readily soluble in water, so that the physical structures of pectin, cellulose and hemicellulose are depolymerized, and the fruit becomes loose^2,3^. Delaying or inhibiting climacteric behavior and reducing the amount of ethylene released is an effective measure to delay the aging and softening of fruit.

1-Aminocyclopropanecarboxylic acid (ACC) oxidase (ACO) catalyzes the conversion of ACC to ethylene, which is the major pathway for the production of endogenous ethylene in fruit. Calcium can inhibit the activity of both ACC synthase (ACS) and ACC oxidase (ACO), and the production of endogenous ethylene. Li *et al*. reported that calcium treatment reduced the respiration rate and ethylene release of netted melon^4^. As an essential plant nutrient, calcium is required for various structural roles in the cell wall and membranes. The study of Chardonnet *et al*. revealed that calcium treatment effectively inhibited the changes of cell wall components to maintain the fruit hardness of Golden Delicious apple^5^. The study of Deng *et al*. showed that calcium treatment reduced the activity and gene expression of cell wall degrading enzymes, to maintain the hardness of grapefruit^6^.

Ethylene can accelerate the ripening process of fruit, and then ripe fruit can release a large amount of ethylene during after-ripening process^7^. 1-Methylcyclopropene (1-MCP) is an inhibitor of ethylene action in plants by competitively binding to ethylene receptor and blocking ethylene signaling, and thus inhibits after-ripening of fruit^8^. The study of Guo *et al*. showed that 1-MCP can decrease the respiratory rate and ethylene release of Yate kiwifruit^9^. 1-MCP can also suppress the activity of cell wall-lysing enzymes, thereby delaying the softening of apple fruits^10^. However, the effects of calcium and 1-MCP on physiological metabolism of melons have been rarely reported.

Therefore, in this study, we investigated the effects of CaCl_2_ and 1-MCP on respiration rate, ethylene release, flesh hardness, decay rate, pectin hydrolase activity and gene expression during storage, using a New Queen melon cultivar Xinmi No. 13 as the experimental material. The results showed that CaCl_2_ and/or 1-MCP can delay the after-ripening and reduce the decay rate of New Queen melons, and better effect can be achieved by using them in combination.

## Results

### Effects of CaCl_2_ and/or 1-MCP on fruit respiration rate and ethylene release

**Figure 1:**
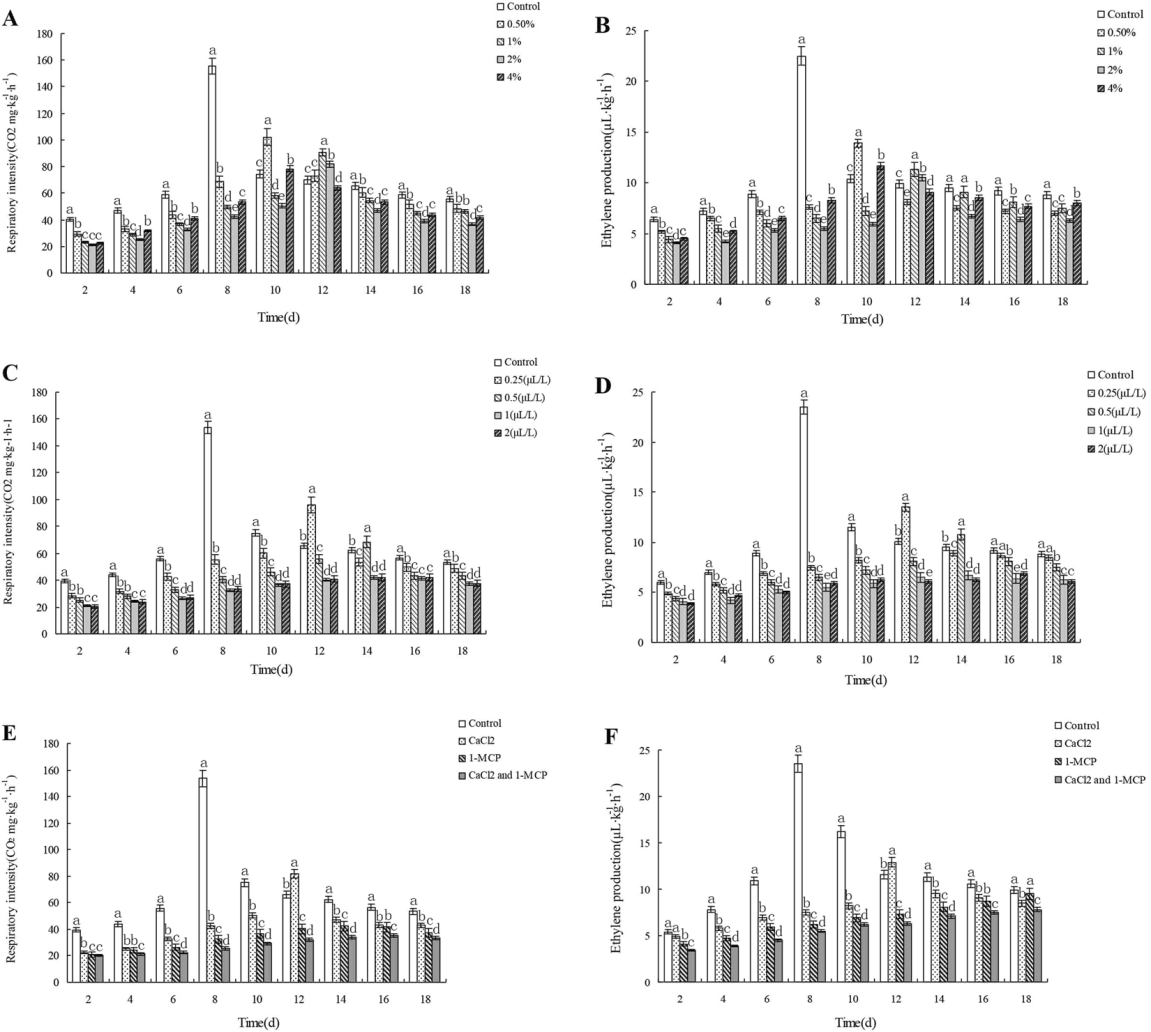
Effects of different concentrations of CaCl_2_ on respiratory rate (**A**) and ethylene release (**B**) during postharvest storage of Xinmi fruits. Effects of different concentrations of 1-MCP on respiratory rate (**C**) and ethylene release (**D**) during postharvest storage of Xinmi fruits. Effects of CaCl_2_ and/or 1-MCP on respiratory rate (**E**) and ethylene release (**F**) during postharvest storage of Xinmi melon. Diferent letters indicate signifcant diferences.

As shown in (Fig. 1A,B), the respiration rate and ethylene release of the fruits treated with 0.045 mol∙L^−1^, 0.09 mol∙L^−1^, 0.18 mol∙L^−1^ or 0.36 mol∙L^−1^ CaCl_2_. The results showed that both the respiration rate and ethylene release of the fruits treated with CaCl_2_ at different concentrations were significantly lower than those of the control (*p* < 0.05). A sharp climacteric peak occurred in both the respiratory rate and ethylene release of the control group on day 8, while the climacteric peaks in all CaCl_2_ treated groups were smaller and appeared later, on day 10 in 0.045 mol∙L^−1^ and 0.36 mol∙L^−1^ CaCl_2_ treated groups, and on day 12 in 0.09 mol∙L^−1^ and 0.18 mol∙L^−1^ CaCl_2_ treated groups. Among all the CaCl_2_ treatments, 0.18 mol∙L^−1^ CaCl_2_ had the lowest respiratory rate and ethylene release, and the smallest climacteric peak value. Moreover, the inhibitory effect of CaCl_2_ on respiration rate and ethylene release increased gradually with CaCl_2_ concentration increasing from 0.045 mol∙L^−1^ to 0.18 mol∙L^−1^, and then decreased when CaCl_2_ concentration was increased to 0.36 mol∙L^−1^. So, 0.18 mol∙L^−1^ was considered as the optimal CaCl_2_ concentration for postharvest treatment of melon fruits.

As shown in (Fig. 1C,D), treatments with 1-MCP at four concentrations all reduced the respiratory rate and ethylene release of melon, and the inhibitory effect of 1-MCP increased in a concentration-dependent manner. The climacteric peaks of respiratory rate and ethylene release appeared on day 12 in 0.25 μL∙L^−1^ 1-MCP treated group, and on day 14 in 0.5 μL∙L^−1^ 1-MCP treated group, respectively. The group treated with 0.5 μL∙L^−1^ 1-MCP had lower respiratory rate and ethylene release, and smaller climacteric peak value than the group treated with 0.25 μL∙L^−1^ 1-MCP (*p* < 0.05). There was no difference in respiratory rate and ethylene release between 1 and 2 μL∙L^−1^ 1-MCP treated groups (*p* > 0.05). And no climacteric peak was observed in these two groups. So, 1 μL∙L^−1^ was considered as the optimal concentration of 1-MCP for treatment of Xinmi fruits. According to the results obtained above, 0.18 mol∙L^−1^ CaCl_2_ and 1 μL∙L^−1^ 1-MCP were used in subsequent experiments.

The effects of CaCl_2_ and/or 1-MCP on the respiratory rate and ethylene release of Xinmi melon were shown in (Fig. 1E,F) The respiratory rate and ethylene release of CaCl_2_ and/or 1-MCP treated groups were lower than those of the control group *(p* < 0.05). There was a climacteric peak in both the respiration rate and ethylene release of the control. In detail, both two indices changed slowly during the first six days of storage, then the respiration rate of the control group increased dramatically from 39.5 CO_2_ mg∙kg^−1^∙h^−1^ on day 6 to 153 CO_2_ mg∙kg^−1^∙h^−1^ on day 8, while ethylene release increased from 5.4 to 23.5 μL∙kg^−1^∙h^−1^. A smaller climacteric peak in respiration rate and ethylene release in CaCl_2_ treated group appeared on day 12 *(p* < 0.05). The 1-MCP treated group had lower respiration rate and ethylene release than the CaCl_2_ treated groups, showing no climacteric changes. The respiration rate and ethylene release of the group treated by CaCl_2_ and 1-MCP in combination were lower than those of all other groups, changed slightly, and showed no climacteric changes.

### Effects of CaCl_2_ on Calcium content in fruits

According to Table 1, total calcium concentration in melons of 0.18 mol calcium treatment was higher than that in the control group, and pectate calcium concentration in the calcium treatment group was also higher than that in the control group, while water-soluble calcium concentration in the treatment group was lower than that in the control group *(p* < 0.05). Ratio of total calcium concentration and other different forms of calcium between 1-MCP treatment and control group showed insignificant differences (*p* > 0.05).

**Table 1:** Effects of different treatments on calcium components of muskmelon flesh. Diferent letters indicate signifcant diferences.

Treatment	Total calcium/(μg·g^−1^)	Pectin-acid calcium (%)	Water soluble calcium (%)
Ca	75.93 ± 0.15^a^	45.93 ± 0.69^a^	19.65 ± 0.71^c^
Ca + 1-MCP	76.06 ± 0.26^a^	46.06 ± 0.11^a^	17.57 ± 0.35^c^
1-MCP	23.15 ± 0.39^b^	25.15 ± 0.27^c^	36.72 ± 0.53^b^
Control	23.32 ± 0.43^b^	29.32 ± 0.36^b^	43.35 ± 0.28^a^

### Effects of CaCl_2_ and/or 1-MCP on fruit hardness

**Figure 2:**
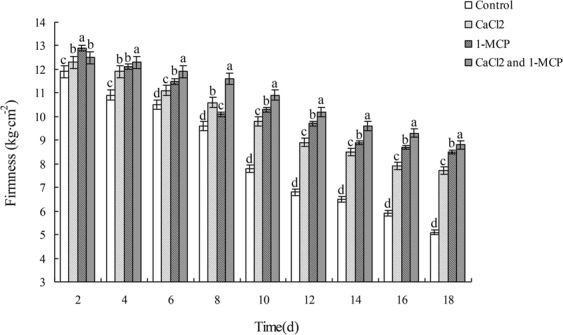
Effects of CaCl_2_ and 1-MCP alone or in combination on fruit hardness during postharvest storage of Xinmi melon. Hardness change of melons in four experiment groups with the time change and differences among the group variations. Diferent letters indicate signifcant diferences.

As shown in (Fig. 2), fruit hardness in all groups decreased continuously over time. Fruit hardness of the control group decreased faster than that of CaCl_2_ and/or 1-MCP treated groups (*p* < 0.05), from 11.9 kg/cm^2^ on day 2 to 5.1 kg/cm^2^ on day 18, and the decrease was steeper from day 8 to day 10. Among all CaCl_2_ and/or 1-MCP treated groups, fruit hardness of CaCl_2_ treated group decreased fastest, followed by that of 1-MCP treated group, and that of the group treated by CaCl_2_ and 1-MCP in combination declined most slowly. Fruit hardness of CaCl_2_ treated group dropped from 12.3 kg/cm^2^ on day 2 to 7.6 kg/cm^2^ on day 18, while that of 1-MCP treated group declined from 12.9 kg/cm^2^ to 8.5 kg/cm^2^, and that of the group treated by CaCl_2_ and 1-MCP together decreased from 12.5 kg/cm^2^ to 8.8 kg/cm^2^.

### Effects of CaCl_2_ and 1-MCP on protopectin and pectin contents

**Figure 3:**
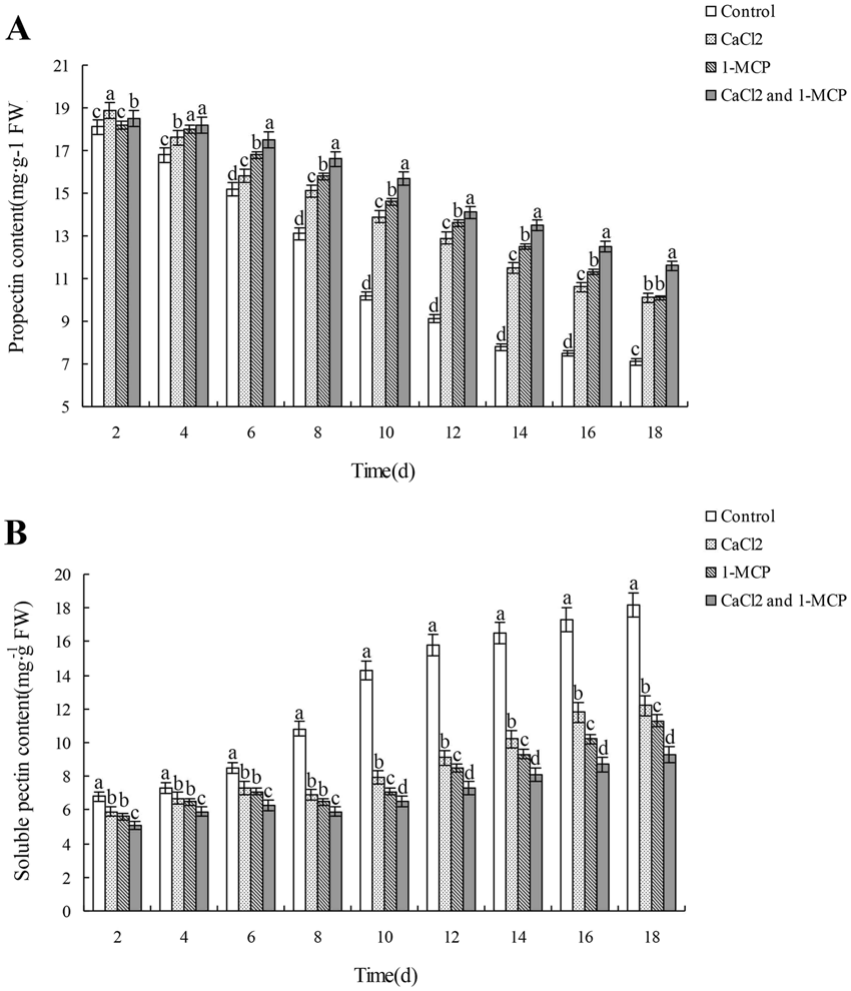
Effects of CaCl_2_ and/or 1-MCP on protopectin (**A**) and soluble pectin (**B**) contents during the storage of Xinmi melon. Change of fruit protopectin concentration (**A**) and soluble pectate concentration (**B**) in four experiment groups with the time change and differences among the group variations. Diferent letters indicate signifcant diferences.

As shown in (Fig. 3A,B), during storage, protopectin content in all groups decreased continuously, while soluble pectin content increased continuously. Protopectin content of the control group decreased from 18.3 mg/g on day 2 to 7.1 mg/g on day 18, and the decrease from day 6 to day 10 was more dramatic; soluble pectin content of the group increased from 6.8 mg/g on day 2 to 18.2 mg/g on day 18, and the increase from day 6 to day 10 was more dramatic. The decrease in protopectin content and the increase in pectin content of all the three CaCl_2_ and/or 1-MCP treated groups were slower than those of the control (*p* < 0.05). Both protopectin content and soluble pectin content changed slightly and showed small difference between CaCl_2_ treated group and 1-MCP-treated group. Among all the groups, the group treated with CaCl_2_ and 1-MCP in combination showed the smallest changes in protopectin content and soluble pectin content, with protopectin decreasing from 18.5 mg/g on day 2 to 11.6 mg/g on day 18, and soluble pectin content increasing from 5.1 to 9.3 mg/g during this period.

### Effects of CaCl_2_ and/or 1-MCP on the activity and gene expression of pectinases

Enzymes are essentially proteins functioning as catalysts, and encoded by genes. So, the expression level of these genes determines the quantity and activity of enzymes, and can be up- or down-regulated by a series of factors.

**Figure 4:**
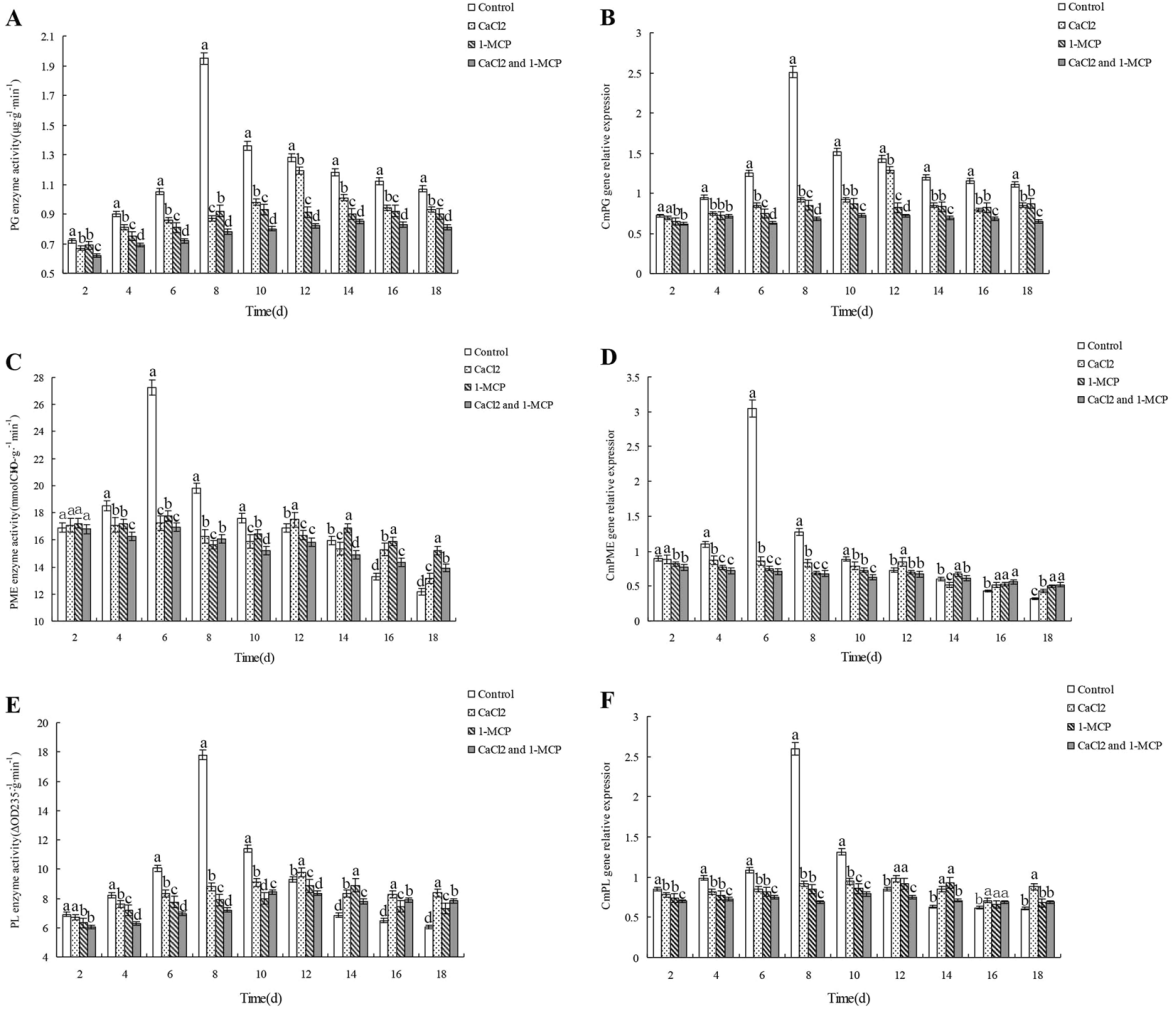
Effects of CaCl_2_ and 1-MCP on PG activity (**A**) and gene expression (**B**), PME activity (**C**) and gene expression (**D**) and PL activity (**E**) and gene expression (**F**) during the storage of Xinmi melon. Change of fruit PG enzyme activity (**A**) and PG enzyme gene expression amount (**B**), PME enzyme activity (**C**) and PME enzyme gene expression amount (**D**), PL enzyme activity (**E**) and PL enzyme gene expression (**F**) and differences among the group variations. Diferent letters indicate signifcant diferences.

As shown in (Fig. 4A,B), PG activity and gene expression of the control group were both higher than those of all CaCl_2_ and/or 1-MCP treated groups (*p* < 0.05). A sharp climacteric peak occurred in PG activity and gene expression of the control group. A smaller climacteric peak in the two indices was observed in CaCl_2_ treated group on day 12, and not in the groups treated with 1-MCP alone or in combination with CaCl_2_. We also found that PG activity and gene expression changed synchronously in every group.

The changes in PME activity and gene expression were shown in (Fig. 4C,D). There was no difference in PME activity between the control group and CaCl_2_ and/or 1-MCP treated groups (*p* > 0.05) during the first several days of storage, and then PME activity of the control group increased dramatically, peaked on day 6, and decreased sharply a level similar to that of CaCl_2_ and/or 1-MCP treated groups from day 12 to day 14, and was lower than that of CaCl_2_ and/or 1-MCP treated groups from day 16 to day 18. PME activity in all CaCl_2_ and/or 1-MCP treated groups varied slowly compared to the control, and gradually decreased over time during storage. Among the three groups, the decrease in PME activity was the greatest in CaCl_2_ treated group, followed by that of 1-MCP treated group, and PME activity in the group treated by CaCl_2_ and 1-MCP in combination declined most slowly.

As shown in (Fig. 4E,F), PL activity and gene expression of the control group were both higher than those of CaCl_2_ and/or 1-MCP treated groups in the first several days, then increased dramatically and peaked on day 8, then decreased sharply, and were lower than those of CaCl_2_ and/or 1-MCP treated groups on day14. Compared with the control group, PL activity and gene expression of CaCl_2_ and/or 1-MCP treated groups changed slightly. The two indices of the group treated by CaCl_2_ alone were higher than those of the groups treated by 1-MCP alone or in combination with CaCl_2_ during the middle stage of storage, and the difference became insignificant during the late stage of storage.

And these enzymes have their own specific correlation with the expression of their enzyme genes. As Fig. 4A,B shows, the highest PG enzyme activity corresponded to the highest amount of enzyme gene expression, while enzyme activity and amount of the enzyme gene expression in other time remained basically the same change tendency, which indicates that the expression amount of PG enzyme gene decided the enzyme activity, and in the primary stage of storage, the increment of enzyme activity was higher than that of the enzyme gene expression amount, it can be therefore concluded that the enzyme consumption rate was lower than the enzyme generation rate. Change tendency of PME enzyme gene was similar to that of its activity, but in the later storage stage the enzyme gene showed smaller amplitude of variation than the enzyme activity did (Fig. 4C,D), therefore in this phase consumption rate of PME enzyme was slightly higher than its generation rate. Figure 4E,F shows the change of PL enzyme activity and its gene expression amount, the enzyme activity increased more than the enzyme gene expression amount did in the primary storage stage, thus the consumption rate of PL enzyme in this phase was lower than the generation rate of enzyme, but the enzyme generation and consumption showed consistency after the climacteric on the 8^th^ day.

### Growth of pathogenic microorganisms in melon flesh of different hardness

**Figure 5:**
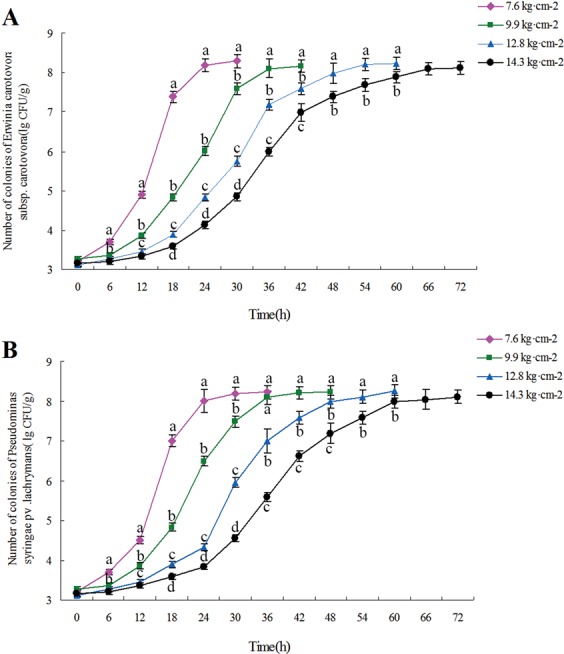
Growth curves of *Pectobacterium carotovora* subsp*. Carotovora* (**A**) and *Pseudomonas syringae* pv. *Lachrymans* (**B**) in flesh of different hardness. In pulp of four hardness degrees, the growth and propagation rate of *Pectobacterium carotovora* subsp*. Carotovora* (**A**) and *Pseudomonas syringae* pv. *Lachrymans* (**B**). Diferent letters indicate signifcant diferences.

The melons of four different hardness values 7.6, 9.9, 12.8 and 14.3 kg∙cm^−2^ were used as samples. Figure (5A,B) show the growth curves of *Pectobacterium* subsp*. Carotovora and Pseudomonas syringae* pv. *Lachrymans* in these samples. These growth curves were typically S-shaped. In addition, the growth rate of the two pathogens decreased with flesh hardness increasing (*p* < 0.05), indicating that softer flesh is more conducive to the growth and preproduction of the two pathogens.

### Effects of CaCl_2_ and 1-MCP on decay rate of melon fruit during storage

**Figure 6:**
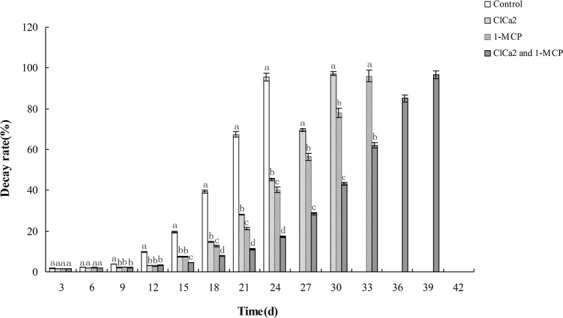
Effects of CaCl_2_ and 1-MCP on decay rate of New Queen’s melon fruit during storage. Rotten rate change of melons in four groups at the storage temperature of 20 °C. Diferent letters indicate signifcant diferences.

Fruit decay is mainly caused by the infection of decay-causing pathogens. The growth and reproduction of microorganisms require suitable environmental conditions. The progressing of postharvest and the variation in fruit hardness also change microbial growth environment during fruit storage. As shown in (Fig. 6), the percentage of rotten fruits of the control group was significantly higher than that of CaCl_2_ and/or 1-MCP treated groups. In addition, the decay rate of the control group was also faster than that of CaCl_2_ and/or 1-MCP treated groups. All the fruits rotted within 27 days in the control group, within 33 days in CaCl_2_ treated group, within 36 days in 1-MCP treated group, and within 42 days in CaCl_2_ + 1-MCP treated group. The difference in decay rate was statistically significant among these groups (*p* < 0.05).

## Discussion

Ethylene is a hormone that can be used to hasten fruit ripening and softening. It can binds to its receptor and accelerates fruit respiratory metabolism, and then up-regulates the expression of pectinase encoding genes, promotes the production of pectinases, which hydrolyze protopectin, and change cell wall structures, and the fruits become soft as a result^11^. In this experiment, there was a positive synergy among respiratory rate, pectinase activity, gene expression and ethylene release in every group.

As an important component of plant cell wall structure, calcium inhibits the activity of cell-wall degrading enzymes. It also has an antagonistic effect on ethylene-induced ripening of fruit^12^. Our data showed that CaCl_2_ treatment significantly reduced the respiration rate, ethylene release and climacteric peak value, and also delayed the climacteric behavior of melon fruit, which was consistent with the findings of Li *et al*. in calcium nitrate treated muskmelon^13^. 1-MCP blocks ethylene binding to its receptor. We found that respiration rate and ethylene release of 1-MCP treated fruits were both significantly lower than those of the untreated fruits, and similar results have been reported by Ma *et al*.^14^. The combined use of CaCl_2_ and 1-MCP can not only antagonizes ethylene action, but also blocks the signal transduction of residual ethylene. In this experiment, we found that better effects in inhibiting respiration rate and ethylene release were achieved when 0.18 mol∙L^−1^ CaCl_2_ and 1 μL∙L^−1^ 1-MCP were used in combination.

Hydrolysis of protopectin into water-soluble pectin is an important cause for fruit softening^15^. The study of Liu *et al*. showed that the content of protopectin decreased gradually during after-ripening of thick-rinded melon, while the content of pectin kept increasing, and the content of cellulose changed little^16^. Based on these results, we investigated the effects of CaCl_2_ and 1-MCP on postharvest ripening and decay of Xinmi melon by measuring the changes in pectin content, pectinase activity and gene expression. The results showed that the content of protopectin in both treated and untreated fruits gradually decreased during storage, while the content of water-soluble pectin increased. Moreover, fruit hardness of the control group declined sharply from day 6 to day 8 of storage, while protopectin was hydrolyzed into pectin at a higher rate, which was consistent to the findings of Jianmei Wei *et al*.^10^.

All PG, PME, PL activity and gene expression of the control group underwent climacteric changes during storage. In detail, the climacteric peaks of PG and PL activity and gene expression occurred on day 8 of storage, when the peaks of respiration rate and ethylene release appeared. The climacteric peaks of PME activity and gene expression occurred earlier on day 6. David A. Brummell *et al*. believed that the hydrolysis of protopectin is initiated by PME at the early stage of storage, and provides substrate for other pectinases such as PG^17^, which may explain why the peaks of PME activity and gene expression occur earlier. PG activity and gene expression of the control group were always higher than those of CaCl_2_ and/or 1-MCP treated groups, but PME and PL activity and gene expression of the control group were lower than those of CaCl_2_ and/or 1-MCP treated groups on day 14 and day 16, respectively. Climacteric for PG activity and gene expression in CaCl_2_ treated group occurred on day 12, but it was not observed in the groups treated by 1-MCP alone or in combination with CaCl_2._ According to the data of pectin content and flesh hardness, we conclude that the climacteric changes in pectinase activity and gene expression play a critical role in hydrolysis of protopectin and fruit softening, and slow changes in them have little effect.

Melon decay is mainly caused by the infection of decay-causing microorganisms. Fruit softening is beneficial to the reproduction and spread of these microorganisms, thus accelerating the decay rate of fruit. Both Cacl_2_ and 1-MCP could inhibit the activity of pectin hydrolase and expression of the enzyme genes, and in this way delay the softening of melons fruit (Fig. 4). Considering the different growth rates of pathogenic microorganisms in fruits of varying hardness (Fig. 5), it can be deduced that CaCl_2_ and 1-MCP can suspend the growth and propagation of microorganism by delaying the fruit softening. So delaying the softening of fruit can decrease the reproduction and spread of microorganisms, thus reducing the decay rate and prolonging the shelf life of fruit.

Pectinase activity and gene expression in the control group were both higher than those of CaCl_2_ and/or 1-MCP treated groups at early storage stage, indicating that the postharvest physiological metabolism of fruits had been inhibited when they were treated with CaCl_2_ and/or 1-MCP. When CaCl_2_ and 1-MCP are used in combination, the mechanisms inhibiting fruit after-ripening are able to integrate and complement each other, thus achieving a better effect in delaying the softening and reducing the decay rate of fruit.

## Materials and Methods

### Plant materials

The seeds of Xinmi No. 13 were provided by the Xinjiang Academy of Agricultural and Reclamation Science, sown from May to June, and transplanted from July to October, 2017, at Shihezi Huayu Seed Breeding Base. Fruits were harvested at maturity and used in this experiment.

### Decay-causing pathogens

Two pathogens were isolated from the rotten New Queen Xinmi melon, *Pectobacterium* subsp. *carotovora* and *Pseudominas syringae* pv. *lachrymans*, and inoculated onto potato dextrose agar (PDA) medium for multiplication.

### Screening of CaCl_2_ and 1-MCP concentrations

The fruits with same weight and shape, and free of defects were selected, Calcium treatment: muskmelons were kept in the 0.045 mol/L, 0.09 mol/L, 0.18 mol/L and 0.36 mol/L CaCl_2_ solution(DSX, China) respectively, experimenter wore rubber gloves and rubbed the muskmelon gently to remove bubbles among the rind wrinkles, and then kept the rind fully exposed to the solution for 25 min, the solution pH was adjusted to 6.5–7.5 using acetic acid, and the same solution without calcium was applied as the control group, then air-dried at room temperature^18^, these treatments were completed within 12 hours after harvest. 1-MCP treatment: muskmelons fumigated with 1.25, 0.5, 1, or 2 μL∙L^−1^ 1-MCP respectively at 20 °C for 24 hours^19^. The optimal concentrations of CaCl_2_ and 1-MCP concentrations were determined according to the respiration rate and ethylene release of the fruits in different treatments during storage.

### Measure calcium concentration

The melons were peeled after 2-day treatment, the epicarp peeled off at the melon equator part, 2-cm thick pulps were taken from the radial direction from outside to inside, to measure the total calcium concentration, water-soluble calcium concentration, and pectate calcium concentration according to Xiao’s method^20^. Specifically, take 10 g homogeneous samples precisely; after smashing and homogenate, extract water-soluble calcium and pectate calcium using deionized water, 1 mol/L calcium chloride, 2% acetic acid and 5% hydrochloric acid in turn. Atomic Absorption Spectrophotometer (AAS) was used to measure the concentration after centrifuging, washing the leaching liquor, getting constant volume with SrCl_2_, drying and digesting.

### Determination of percentage of rotten fruits

A total of 400 fruits with same weight and shape, are synchronous in maturity and free of defects, were selected as the samples, randomly and equally divided into four groups, with 100 fruits in each group. One of the groups, untreated with CaCl_2_ or 1-MCP, was used as the control, and the other three groups were treated with CaCl_2_ and 1-MCP alone or in combination, within 12 h after harvesting, at their optimal concentrations determined above. Then, the fruits were bagged with nylon net bags, and packaged in carton boxes, with ten fruits in each box, and stored in a cellar at a temperature of (20 ± 1) °C and at a relative humidity of (50 ± 5)%.

Rotter fruits were counted once every 10 days, and the percentage of rotten fruits was calculated with the formula as follows:

$${\rm{Percentage}}\,{\rm{of}}\,{\rm{rotten}}\,{\rm{fruits}}\,( \% )=[{\rm{Number}}\,{\rm{of}}\,{\rm{rotten}}\,{\rm{fruits}}/{\rm{Total}}\,{\rm{number}}\,{\rm{of}}\,{\rm{tested}}\,{\rm{fruits}}]\times 100$$

### Measurement of fruit respiration rate, ethylene release and hardness

In order to measure respiration rate, the fruits were placed in the sample container of 3051 H respirometer (LV BO, China). The gas flow rate was 0.4 L∙min^−1^. Nine fruits were tested in each measurement, and each measurement was repeated three times. The resulting respiration rate was expressed in CO_2_ mg∙kg^−1^∙h^−1^ ^21^.

The amount of ethylene released was measured using a Trace GC 1300 gas chromatograph (Thermo USA) according to the method of Li *et al*.^22^. Nine fruits were tested in each measurement, and each measurement was repeated three times.

Ethylene release was calculated using the formula as follows:

$${\rm{Ethylene}}\,{\rm{release}}\,({\rm{\mu }}L\cdot {{\rm{kg}}}^{-1}\cdot {{\rm{h}}}^{-1})=(C\times V)/(m\times t\times 1000)$$

Where, *C* is the amount of ethylene released by samples (μL∙L^−1^), *V* is difference between the volume of dryer space and the volume of the samples (mL), *m* is the weight of the samples (kg), and *t* is time (h).

Fruit hardness (kg∙cm^−2^) was tested using an AGY-1 texture analyzer (AI LI, China). Nine fruits were analyzed in each measurement, and every measurement was repeated three times^23,24^.

### Detection of protopectin and soluble pectin contents

The contents of protopectin and pectin in every gram of sample (mg∙g^−1^ FW) were measured using carbazole-sulfuric acid method with the method of Qi *et al*.^25^.

### Pectinase activity analysis

Polygalacturonase (PG) activity was measured as previously described^26^. One unit of PG activity was defined as the amount of enzyme releasing 1 μmol of galacturonic acid per minute by decomposing polygalacturonic acid in one gram of fresh sample at 27 °C (μg∙g^−1^∙min^−1^). Pectin methylesterase (PME) activity was quantified by NaOH titration^27,28^. One unit of PME activity was defined as the amount of enzyme releasing 1 mmol of CH3O- from one gram of fresh sample (mmol CH3O-∙g^−1^∙min^−1^. One unit of pectin lyase (PL) activity was defined as the amount of enzyme needed to cause an increase in optical density OD235 by 1 per minute using one gram of fresh sample (ΔOD235∙g^−1^∙min^−1^)^29^.

### Quantitative RT-PCR analysis

Total RNA was isolated from melon flesh using the EasyPure Plant RNA Kit (TRANS, Beijing, China); RNA quality was examined on agarose gels and quantified on a NanoDrop (Thermo) photometer. The RNA isolation for gene expression was obtained of three biological replicates. All RNA samples were treated with RNase free DNase I (Ambion) to remove contaminant DNA traces. To amplify the selected genes, cDNA was amplified by PCR using the following primers were listed in (Table 2) and synthesized with the EasyScript First-Strand cDNA Synthesis SuperMix (TRANS, Beijing, China). Amplifcation was carried out through initial denaturation at 94 °C for 2 min, followed by 38 cycles of denaturation at 94 °C for 30 s, annealing at 57 °C for 30 s, and elongation at 72 °C for 2 min. The PCR products from each amplifcation reaction were separated on 2.5% (w/v) agarose gels. Real-time quantification RT-PCR reactions were performed in Bio-RAD MyiQTM Real-time PCR Detection System (Bio-Rad, USA) using the TransStart Top Green qPCR SuperMix (TRANS, Beijing, China) according to the manufacturer’s instructions^30,31^.

**Table 2:** Primers for qRT-PCR analysis.

Gene name	Gene accession No	Primer sequence
CmPG	MELO3C000985	F5′-TTGACAGCGTTGGTTACCTG-3′
		R5′-CCACATAACTCAGTCCCCAA-3′
CmPME	MELO3C024917	F5′-GATTTGCGTGTGGATGTTTG-3′
		R5′-CCGGAGCAATTTTTATCCAC-3
CmPL	MELO3C002431	F5′-CCAGATTTCCCAGGCTTACA-3′
		R5′-TGGGGCTAAAGTGGAAATTG-3′
CmActin	MELO3C011913	F5′-CCAAAGGCTGCAAGAATAGC-3′
		R5′-TTTGACCTTTGGGTGGGTAG-3′

### Bacterial culture and counting

The flesh samples of melons of four different hardness values were cut into cubes of 1.5 cm^3^. *Pectobacterium* subsp. *carotovora* and *Pseudominas syringae* pv. *lachrymans* were separately inoculated into the flesh cubes, and cultured in an incubator at 27 °C and a relative humidity of 60%, and the number of bacteria was counted once every 6 hours.

*Pectobacterium* subsp*. Carotovora* and *Pseudomonas syringae* pv. *Lachrymans* obtained in the separation were cultured on PDA slant medium at 27°C for 7 days. The bacteria was diluted with the sterile water, oscillated fully and shaken well, calculated using microscope, to make sure the concentration of the bacterial suspension up to 10^6^ CFU/mL. Then 50 pieces of pulp and 4 melons of different hardness were taken and, sliced into smaller ones, 1.5 cm^3^ each, 2 μL bacterial suspension sucked precisely with pipette and each pulp inoculated with the suspension. Then cultured in an incubator at 27 °C and a relative humidity of 60%, and the number of bacteria was counted once every 6 hours. In detail, the fruit flesh was weighed, smashed, and filtered through a 60-mesh filter, spread in a square of 1 cm × 1 cm on a slide, heated in boiling water for 10 minutes, air dried, then stained with 0.1% methylene blue for 2 minutes. Subsequently, the number of bacteria in 20 random fields was counted under an oil-immersion microscope, and their average was calculated. Finally, the growth curves of *Pectobacterium* subsp. *carotovora* and *Pseudominas syringae* pv. *lachrymans* in melons of different hardness values were plotted based on these data^32^.

### Statistical analysis

Excel statistics and plotting were used to analyze the experimental data. SPSS17.0 software system was used to analyze the variance and *p* < 0.05 was considered as statistically significant.
